# Convolutional neural network-based system for endocytoscopic diagnosis of early gastric cancer

**DOI:** 10.1186/s12876-022-02312-y

**Published:** 2022-05-12

**Authors:** Hiroto Noda, Mitsuru Kaise, Kazutoshi Higuchi, Eriko Koizumi, Keiichiro Yoshikata, Tsugumi Habu, Kumiko Kirita, Takeshi Onda, Jun Omori, Teppei Akimoto, Osamu Goto, Katsuhiko Iwakiri, Tomohiro Tada

**Affiliations:** 1 Department of Gastroenterology, Nippon Medical School Hospital, 1-1-5, Sendagi, Bunkyo-ku, Tokyo, 113-8603 Japan; 2 Tada Tomohiro Institute of Gastroenterology and Proctology, Saitama, Japan; 3 AI Medical Service Inc., Tokyo, Japan

**Keywords:** Artificial intelligence, Convolutional neural network, Endocytoscopy, Gastric cancer, Gastroenterology, Early detection of cancer

## Abstract

**Background:**

Endocytoscopy (ECS) aids early gastric cancer (EGC) diagnosis by visualization of cells. However, it is difficult for non-experts to accurately diagnose EGC using ECS. In this study, we developed and evaluated a convolutional neural network (CNN)-based system for ECS-aided EGC diagnosis.

**Methods:**

We constructed a CNN based on a residual neural network with a training dataset comprising 906 images from 61 EGC cases and 717 images from 65 noncancerous gastric mucosa (NGM) cases. To evaluate diagnostic ability, we used an independent test dataset comprising 313 images from 39 EGC cases and 235 images from 33 NGM cases. The test dataset was further evaluated by three endoscopists, and their findings were compared with CNN-based results.

**Results:**

The trained CNN required 7.0 s to analyze the test dataset. The area under the curve of the total ECS images was 0.93. The CNN produced 18 false positives from 7 NGM lesions and 74 false negatives from 28 EGC lesions. In the per-image analysis, the accuracy, sensitivity, specificity, positive predictive value (PPV), and negative predictive value (NPV) were 83.2%, 76.4%, 92.3%, 93.0%, and 74.6%, respectively, with the CNN and 76.8%, 73.4%, 81.3%, 83.9%, and 69.6%, respectively, for the endoscopist-derived values. The CNN-based findings had significantly higher specificity than the findings determined by all endoscopists. In the per-lesion analysis, the accuracy, sensitivity, specificity, PPV, and NPV of the CNN-based findings were 86.1%, 82.1%, 90.9%, 91.4%, and 81.1%, respectively, and those of the results calculated by the endoscopists were 82.4%, 79.5%, 85.9%, 86.9%, and 78.0%, respectively.

**Conclusions:**

Compared with three endoscopists, our CNN for ECS demonstrated higher specificity for EGC diagnosis. Using the CNN in ECS-based EGC diagnosis may improve the diagnostic performance of endoscopists.

## Background

Gastric cancer is the fifth most common cancer and the third leading cause of cancer death worldwide [1]. Esophagogastroduodenoscopy (EGD) enables a more accurate diagnosis of early gastric cancer (EGC); therefore, the population-based EGD screening was introduced in Japan, which aimed to reduce gastric cancer (GC) mortality [2]. However, conventional white light imaging endoscopy (WLE) misses a significant number of EGCs [3]. To overcome the limitations associated with WLE, image-enhanced endoscopy such as narrow-band imaging (NBI) has been developed with better diagnostic accuracy than WLE [4]. Previous reports have shown that NBI was useful for EGC diagnosis, and magnifying endoscopy with NBI (ME-NBI) was more accurate than WLE [5–7]. Despite improvements in EGD diagnosis, forceps biopsy is still required for histopathological diagnosis and is the gold standard of GC diagnosis. However, forceps biopsy has limitations, including restrictions due to antithrombotic medicine taken by the patient [8], sampling error caused by mistargeting [9], complications after biopsy [10], or additional medical cost [11].

Endocytoscopy (ECS), a contact-type ultrahigh-magnification endoscopy, directly observes gastrointestinal mucosal cells and neoplastic cells in real time. ECS achieves more accuracy as an optical biopsy approach and can allow endoscopists to skip forceps biopsy in some instances [12–16]. We have previously shown that ECS showed satisfactory accuracy for EGC diagnosis [17], and adequate training leads to a good concordance rate of ECS diagnosis regardless of endoscopic expertise [18]. Sufficient training by an appropriate expert instructor is required to obtain an excellent ECS diagnosis for EGC.

In recent years, artificial intelligence (AI), and the deep learning subtype with a convolutional neural network (CNN) in particular, has been developed as a supportive tool to expand human intelligence and problem-solving ability in the medical field [19]. Deep learning has been adopted for image recognition and is suitable for clinical application, especially for diagnoses made in the fields of radiology [20], pathology [21], and gastrointestinal endoscopy [22]. In deep learning, the AI machine itself creates effective patterns by extracting and learning features that are difficult for humans to define, which improved the machine’s ability to recognize the image [23]. In the diagnosis of GC, the usefulness of the computer-aided diagnosis (CAD) systems, which use a wide variety of endoscopic images such as white light imaging (WLI), NBI, and flexible spectral imaging color enhancement (FICE), has been reported [24–28]. For ME-NBI in EGC diagnosis, some of these studies have reported a sensitivity from 91.2 to 95.4% and specificity from 71.0 to 90.6% [27, 28].

In the present study, we developed a CNN-based system on ECS images of EGC, investigated its diagnostic ability in ECS of diagnosis of EGC, and compared the ability to that of endoscopists.

## Methods

### Study subjects

All ECS images were retrospectively collected from patients who underwent ECS for the diagnosis of EGC at Nippon Medical School Hospital (Tokyo, Japan). Exclusion criteria were as follows: (1) presence of advanced GC; (2) diffuse-type GC; (3) presence of ulcer or ulcer scar; (4) presence of benign polyps such as a foveolar hyperplastic polyp or fundic gland polyp; and (5) poor-quality images caused by halation, bleeding, mucus, defocus, and poor staining. Finally, a total of 2171 ECS images from 198 lesions of 130 patients were analyzed in this study. All 130 patients had EGC and had undergone endoscopic submucosal dissection. All ECS images were extracted in JPEG format. This study was conducted in accordance with the Declaration of Helsinki. The study protocol with opt-out consent was approved by the medical ethics committee of the Nippon Medical School Hospital (registry no. 30-08-984). All data were fully anonymized prior to analysis to protect patient privacy.

### ECS observation

All ECS procedures were performed with GIF-Y0002, GIF-Y0074, and GIF-H290EC (Olympus Co., Tokyo, Japan) and the video processors EVIS LUCERA CV-260/CLV-260 or EVIS LUCERA ELITE CV-290/CIV-290SL (Olympus Co., Tokyo, Japan) in the present study. All procedures were performed by two experienced endoscopists as preoperative screenings of endoscopic submucosal dissection (ESD). As an observation protocol, the part of interest was observed by white light, NBI, and magnified NBI observation. After the magnified NBI observation, we performed vital staining and started ECS observation. For in vivo dyeing, double staining with crystal violet and methylene blue was used. First, we observed the background noncancerous mucosa around the cancer and then the cancerous area. When observing a cancerous area, we started from a part that is clearly cancerous mucosa by other observation methods and moved around the observation site while remaining in contact with the lesion. If the lesion was considered to have moved outside of the lesion or if we recognized clear boundaries in cancerous mucosa, endocytoscopy was moved away from the lesion and the observation was repeated. ECS images were obtained either from EGC or noncancerous gastric mucosa (NGM). All EGCs and NGM surrounding the cancer were resected via ESD, and the final histological diagnoses were identified. ECS images were obtained either from EGC or NGM. All EGCs and NGM surrounding the cancer were resected via ESD, and the final histological diagnoses were identified.

### Preparation training and test data sets

**Fig. 1:**
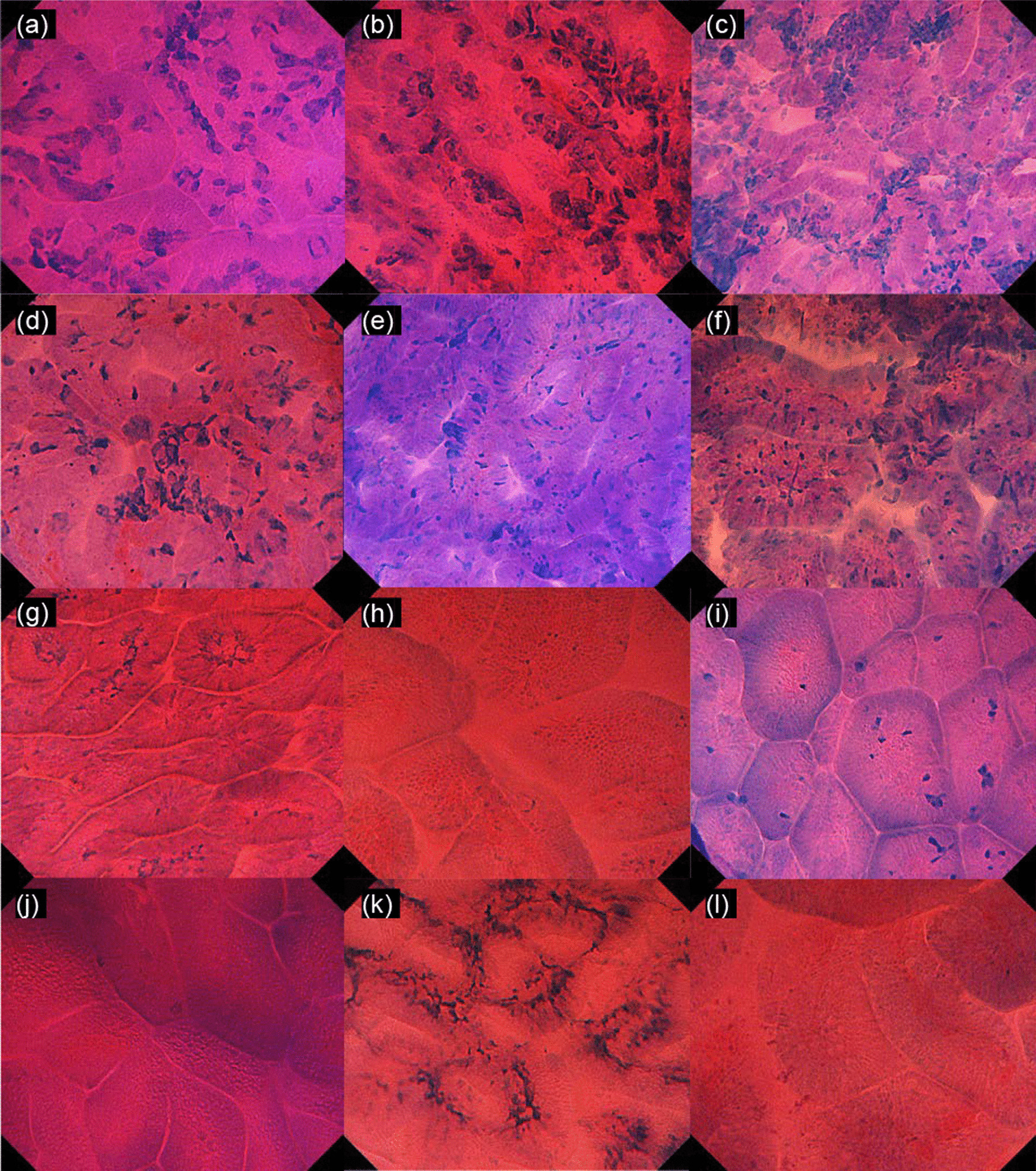
Representative endocytoscopic images in the training dataset. **a**–**f** Cases of intestinal-type early gastric cancer showing specific irregularities in gland structure and cell nuclei. **g**–**l** Cases of noncancerous gastric mucosa a, in which the gland lumen is well preserved and mucosal cells are regularly arranged

All images were reviewed by one endoscopist (H.N.) and classified as EGC or NGM. In addition, we divided all images into training, validation, and test datasets by random selection as follows: (1) the training, validation, and test datasets were mutually exclusive; (2) ECS images in a single patient were not divided into training, validation, and test datasets; (3) the total number of images for training and validation datasets was set to 3 times the number of images for the test dataset. For the training and validation datasets, we collected 906 images from 61 EGCs (Fig. 1a–f) and 717 images from 65 NGM (Fig. 1g–l). Moreover, we prepared a test dataset of ECS images, which included 313 images of 39 EGCs and 235 images of 33 NGMs. (4) The validation dataset was used for internal validation in construction of CNN. (5) The training and validation data were divided randomly by engineers of AI Medical Service Inc.

### Constructing CNN models

A PyTorch was employed as the deep learning framework. Our AI system was created using ResNet50, which is one of the models for image recognition; 812 cancer images and 644 noncancer images of ECS were used to train the AI system. For the validation, 94 cancer images and 73 noncancer images were used. No cross-validation was performed in this study. Stochastic gradient descent was used as the optimization function for training with a learning rate of 0.0001, moments of 0.9, and weight decay of 0.000005. The batch size was set to 5, and the number of epochs was set to 100 for training. The image was preprocessed by resizing it to 256 × 256 pixels and cropping the center to 224 × 224 pixels so that the corners of the image would not affect the inference of the AI model. The model created was 91 epochs, which had the highest accuracy in the validation data.

### Outcome measures

#### Per-image analysis

After constructing the CNN, we evaluated the diagnostic ability through the test dataset. For each image, the CNN constructed the probability score for EGC and receiver operating characteristic (ROC) curve by varying the operating threshold. The area under the curve (AUC) was calculated using the ROC curve. The cut-off value was determined as 0.50. As shown in Table 1, the accuracy, sensitivity, specificity, positive predictive value (PPV), and negative predictive value (NPV) of cancer diagnosis of the CNN in the per-image analysis were measured; the parameters are defined in Table 1. Some images in the test dataset were analyzed with a heatmap obtained by applying Gradient-weighted Class Activation Mapping (Grad-CAM) to the trained CNN [29].

**Table 1:** Definition of accuracy, sensitivity, specificity, PPV, and NPV in the per-image analysis (per-lesion analysis)

Parameter	Definition
Accuracy	Correctly diagnosed images (lesions) by the CNN or endoscopists/total images (lesions)
Sensitivity	Correctly diagnosed EGC images (lesions) by the CNN or endoscopists /total EGC images (lesions)
Specificity	Correctly diagnosed NGM images (lesions) by the CNN or endoscopists /total NGM images (lesions)
PPV	Correctly diagnosed images (lesions) by the CNN or endoscopists/total images (lesions) diagnosed as EGC by the CNN or endoscopists
NPV	Correctly diagnosed EGC images (lesions) by the CNN or endoscopists /total images (lesions) diagnosed as NGM by the CNN or endoscopists

PPV, positive predictive value; NPV, negative predictive value; CNN, convolutional neural network; EGC, early gastric cancer; NGM, noncancerous gastric mucosa

#### Per-lesion analysis

When more than half of ECS images from one lesion were classified as EGC, the lesion was defined as EGC. We calculated the accuracy, sensitivity, specificity, PPV, and NPV of cancer diagnosis of the CNN in the per-lesion analysis as well as per-image analysis (Table 1).

### Diagnostic performance: CNN versus endoscopists

We compared the diagnostic ability of the CNN with three endoscopists blinded to the histological and CNN diagnoses and who independently reviewed the same test dataset. Of the three endoscopists, two endoscopists (endoscopist A [K.H.] and endoscopist B [E.K]) were experienced endoscopists, with > 5 years’ experience in endoscopy, and one endoscopist (endoscopist C [K.Y.]) was a trainee, with < 2 years’ experience. We classified all images as EGC or NGM. Before the review, the endoscopists were informed of the diagnostic criteria of ECS for GC based on high-grade ECS atypia, which were previously described [17, 18], using a training set composed of a schema, a pathological image, and 10 ECS images of each EGC and NGM. After review, we calculated the accuracy, sensitivity, specificity, PPV, and NPV of cancer diagnosis in the per-image analysis of both the endoscopists and CNN.

### Statistical analyses

All analyses were performed using the EZR software program (Saitama Medical Center, Jichi Medical University) [30]. Fisher’s exact test was used for comparisons between the endoscopists and CNN. *P* < 0.05 was considered statistically significant.

## Results

### Clinicopathological features of EGCs in the test dataset

Thirty-nine EGCs of 38 patients were enrolled in test datasets, and all EGCs were resected via ESD. Among the 39 patients, 3 patients underwent additional surgical treatment of gastrectomy according to Japanese GC treatment guidelines [31]. Twenty-six patients (68.4%) were males, and 12 patients (31.6%) were females. The median age was 77 years (range, 53–91 years). The most frequent location and macroscopic type were lower-third (18/39 patients; 46.1%) and 0-IIc type (27/39 patients; 69.2%). The median diameter of EGCs was 18 mm (range, 5–49 mm). Only three cases (7.7%) were submucosal invasive cancer (pT1b) according to JGCA.

### Per-image analysis

**Fig. 2:**
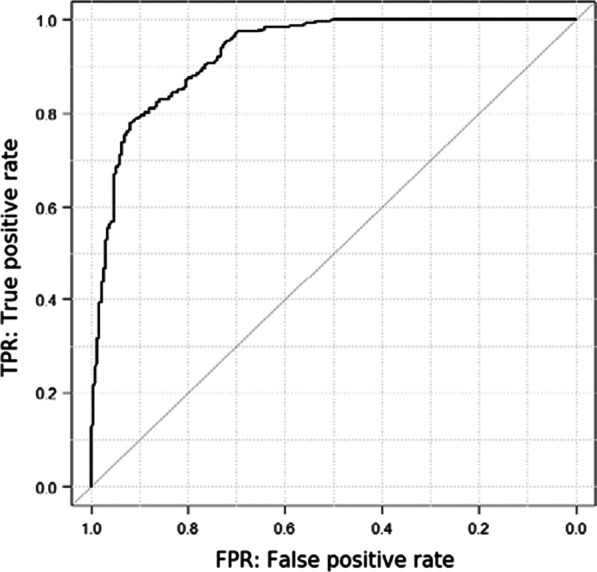
Receiver operating characteristics curve for the artificial intelligence system. The area under the curve was 0.93

**Fig. 3:**
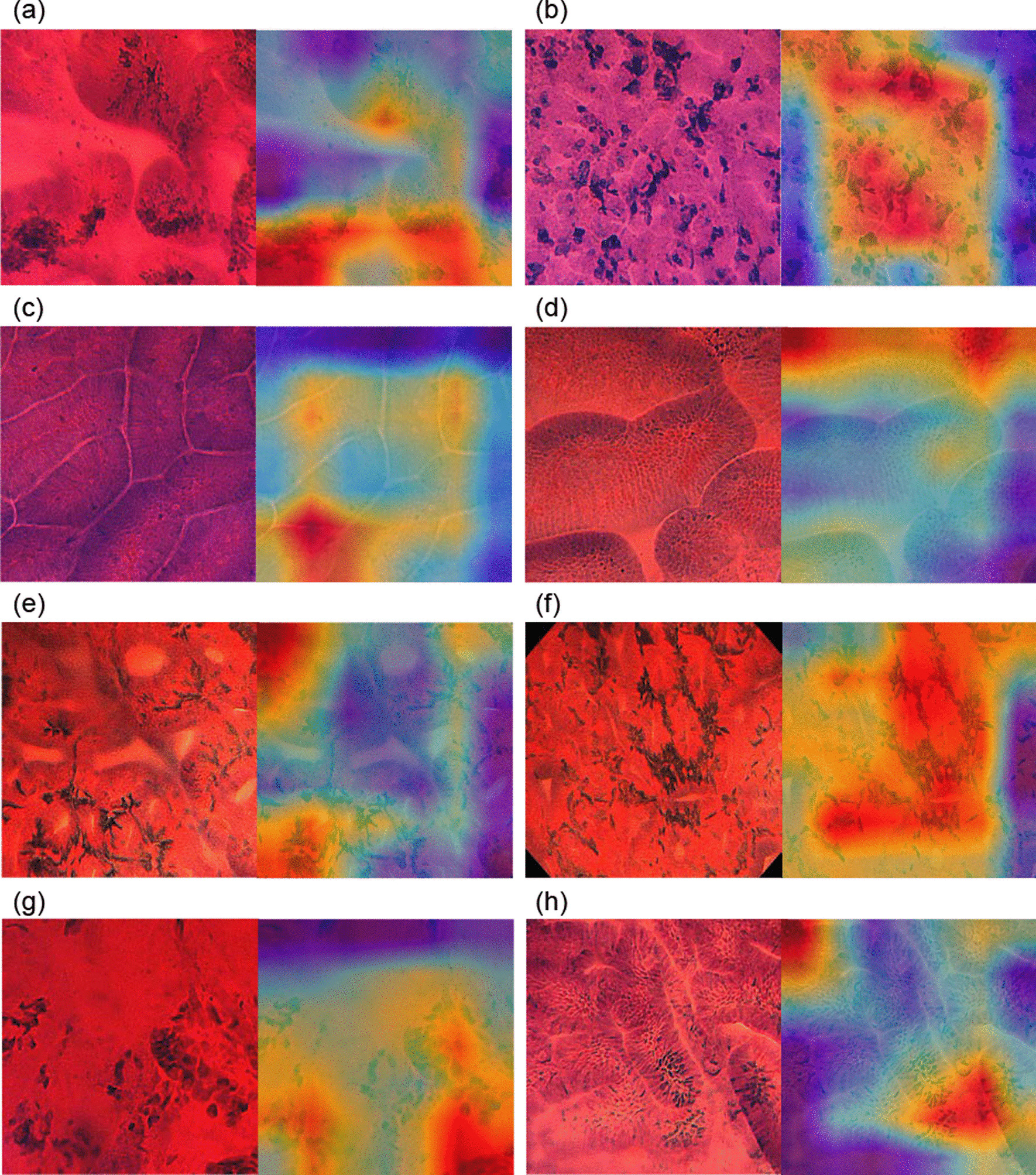
Endocytoscopic images with heatmap in the test dataset. **a**, **b** Cases of intestinal-type early gastric cancer correctly diagnosed by the convolution neural network (CNN). **c**, **d** Noncancerous gastric mucosa correctly diagnosed by the CNN. **e**, **f** False-positive cases. **g**, **h** False-negative cases

The trained CNN required 7.0 s to analyze the test dataset. Based on the probability score for EGC, the AUC was 93.0% (Fig. 2) and the cut-off value for the probability score was 0.5. The accuracy of the CNN for EGC was 83.2%, with 456 of 548 images being correctly diagnosed (Table 2). The sensitivity, specificity, PPV, and NPV for EGC diagnosis by the CNN were 76.4%, 92.3%, 93.0%, and 74.6%, respectively. Representative images of ECS with heatmap visualizations in EGC that were correctly diagnosed by the CNN, NGM correctly diagnosed by the CNN, and false-positive and negative cases are shown in Fig. 3. In the EGC images, the irregular and swelling nuclei, displayed in red color, were determined as cancer by the CNN. In the NGM images, the CNN may focus on regularly lined cells and wide lumens displayed in yellow and red colors.

**Table 2:** Diagnostic performances of CNN and endoscopists

		Per–image	Per–lesion
Accuracy, % (95% CI)	CNN	83.2 (79.8–86.2)	86.1 (75.9–93.1)
	Endoscopists	76.8 (74.6–78.8)	82.4 (76.7–87.2)
Sensitivity, % (95% CI)	CNN	76.4 (71.3–81.0)	82.1 (66.5–92.5)
	Endoscopists	73.4 (70.4–76.2)	79.5 (71.0–86.4)
Specificity, % (95% CI)	CNN	92.3 (88.2–95.4)	90.9 (75.7–98.1)
	Endoscopists	81.3 (78.2–84.1)	85.9 (77.4–92.0)
PPV, % (95% CI)	CNN	93.0 (89.2–95.8)	91.4 (76.9–98.2)
	Endoscopists	83.9 (81.2–86.4)	86.9 (79.0–92.7)
NPV, % (95% CI)	CNN	74.6 (69.2–79.5)	81.1 (64.8–92.0)
	Endoscopists	69.6 (66.4–72.8)	78.0 (69.0–85.4)

CNN, convolutional neural network; CI, confidence interval; PPV, positive predictive value; NPV, negative predictive value; AUC, area under the curve

### Misdiagnosed images by the CNN

Eighteen images from 7 NGM lesions were identified as false-positive by the CNN, probably because of overstaining, mucus, and defocus. All 18 images were misdiagnosed as EGC by at least one endoscopist. Seventy-four images from 28 EGC lesions were identified as false-negative by the CNN, probably due to insufficient staining and defocus. As shown in Fig. 3, comma-shaped cells in the interstitial portion of gastric glands were misdiagnosed as cancer cells by the CNN, probably because these cells were hyperchromatic and similar to the nuclei of cancer cells. In the false-negative cases, the CNN focused on the area where the lumen and nuclei were unclear due to heterogeneous staining.

### Per-lesion analysis

A total of 39 EGC lesions and 33 NGM lesions were included in the study. Thirty-two lesions were correctly diagnosed as GC by the CNN (sensitivity, 82.1%). The CNN accurately diagnosed 30 of 33 NGM lesions as noncancerous (specificity, 90.9%). The overall accuracy, PPV, and NPV for EGC diagnosis by the CNN were 86.1%, 91.4%, and 81.1%, respectively (Table 2).

### Diagnostic performance: CNN versus endoscopists

Comparison of the diagnostic performances between the CNN and endoscopists in the per-image analysis is summarized in Table 2. It took > 20 min for the endoscopists to review all the images. The overall accuracy, sensitivity, specificity, PPV, and NPV for EGC diagnosis by the three endoscopists were 76.8%, 73.4%, 81.3%, 83.9%, and 69.6%, respectively. On comparing between experienced endoscopists and CNN, sensitivity was significantly higher for endoscopists and specificity was significantly higher for CNN. No significant difference in accuracy was noted between two experienced endoscopists and the CNN (Tables 3, 4). On comparing between trainee and CNN, CNN was superior to the trainee (Table 5). The specificity for EGC diagnosis by the CNN was significantly higher than those by the endoscopists. In the per-lesion analysis, the overall accuracy, sensitivity, specificity, PPV, and NPV for EGC diagnosis by the two endoscopists were 82.4%, 79.5%, 85.9%, 86.9%, and 78.0%, respectively (Table 5). No significant differences in the per-lesion diagnostic performance was observed between the CNN and two experienced endoscopists; however, the CNN was superior to the trainee regarding accuracy and sensitivity (Tables 6, 7, 8).

**Table 3:** Diagnostic performances of CNN and endoscopist A in the per-image analysis

	CNN	Endoscopist A	*P* value
Accuracy, % (95% CI)	83.2 (79.8–86.2)	82.3 (78.8–85.4)	0.75
Sensitivity, % (95% CI)	76.4 (71.3–81.0)	86.6 (82.3–90.2)	0.0014
Specificity, % (95% CI)	92.3 (88.2–95.4)	76.6 (70.7–81.9)	< 0.001
PPV, % (95% CI)	93.0 (89.2–95.8)	83.1 (78.6–87.0)	< 0.001
NPV, % (95% CI)	74.6 (69.2–79.5)	81.1 (75.3–86.0)	0.089

CNN, convolutional neural network; CI, confidence interval; PPV, positive predictive value; NPV, negative predictive value; AUC, area under the curve

**Table 4:** Diagnostic performances of CNN and endoscopist B in the per-image analysis

	CNN	Endoscopist B	*P* value
Accuracy, % (95% CI)	83.2 (79.8–86.2)	84.1 (80.8–87.1)	0.75
Sensitivity, % (95% CI)	76.4 (71.3–81.0)	83.4 (78.8–87.3)	0.036
Specificity, % (95% CI)	92.3 (88.2–95.4)	85.1 (79.9–89.4)	0.019
PPV, % (95% CI)	93.0 (89.2–95.8)	88.2 (83.9–91.6)	0.063
NPV, % (95% CI)	74.6 (69.2–79.5)	79.4 (73.8–84.2)	0.74

CNN, convolutional neural network; CI, confidence interval; PPV, positive predictive value; NPV, negative predictive value; AUC, area under the curve

**Table 5:** Diagnostic performances of CNN and endoscopist C in the per-image analysis

	CNN	Endoscopist C	*P* value
Accuracy, % (95% CI)	83.2 (79.8–86.2)	63.9 (59.7–67.9)	< 0.001
Sensitivity, % (95% CI)	76.4 (71.3–81.0)	50.2 (44.5–55.8)	< 0.001
Specificity, % (95% CI)	92.3 (88.2–95.4)	82.1 (76.6–86.8)	0.001
PPV, % (95% CI)	93.0 (89.2–95.8)	78.9 (72.6–84.3)	< 0.001
NPV, % (95% CI)	74.6 (69.2–79.5)	55.3 (49.9–60.6)	< 0.001

CNN, convolutional neural network; CI, confidence interval; PPV, positive predictive value; NPV, negative predictive value; AUC, area under the curve

**Table 6:** Diagnostic performances of CNN and endoscopist A in the per-lesion analysis

	CNN	Endoscopist A	*P* value
Accuracy, % (95% CI)	86.1 (75.9–93.1)	87.5 (77.6–94.1)	1
Sensitivity, % (95% CI)	82.1 (66.5–92.5)	94.9 (82.7–99.4)	0.15
Specificity, % (95% CI)	90.9 (75.7–98.1)	78.8 (61.1–91.0)	0.30
PPV, % (95% CI)	91.4 (76.9–98.2)	84.1 (69.9–93.4)	0.50
NPV, % (95% CI)	81.1 (64.8–92.0)	92.9 (76.5–99.1)	0.28

CNN, convolutional neural network; CI, confidence interval; PPV, positive predictive value; NPV, negative predictive value; AUC, area under the curve

**Table 7:** Diagnostic performances of CNN and endoscopist B in the per-lesion analysis

	CNN	Endoscopist B	*P* value
Accuracy, % (95% CI)	86.1 (75.9–93.1)	88.9 (79.3–95.1)	0.80
Sensitivity, % (95% CI)	82.1 (66.5–92.5)	89.7 (75.8–97.1)	0.52
Specificity, % (95% CI)	90.9 (75.7–98.1)	87.9 (71.8–96.6)	1
PPV, % (95% CI)	91.4 (76.9–98.2)	89.7 (75.8–97.1)	1
NPV, % (95% CI)	81.1 (64.8–92.0)	87.9 (71.8–96.6)	0.52

CNN, convolutional neural network; CI, confidence interval; PPV, positive predictive value; NPV, negative predictive value; AUC, area under the curve

**Table 8:** Diagnostic performances of CNN and endoscopist C in the per-lesion analysis

	CNN	Endoscopist B	*P* value
Accuracy, % (95% CI)	86.1 (75.9–93.1)	70.8 (58.9–81.0)	0.042
Sensitivity, % (95% CI)	82.1 (66.5–92.5)	53.8 (37.2–69.9)	0.014
Specificity, % (95% CI)	90.9 (75.7–98.1)	90.9 (75.7–98.1)	1
PPV, % (95% CI)	91.4 (76.9–98.2)	87.5 (67.6–97.3)	0.68
NPV, % (95% CI)	81.1 (64.8–92.0)	62.5 (47.4–76.0)	0.092

CNN, convolutional neural network; CI, confidence interval; PPV, positive predictive value; NPV, negative predictive value; AUC, area under the curve

## Discussion

In this study, we constructed an AI-assisted ECS diagnosis system for EGC using CNN. On comparing accuracy, no significant difference was found between the CNN and two experienced endoscopists. In contrast, the CNN had a significantly higher rate of accuracy than the trainee. Using the CNN, ECS diagnosis for EGC may be leveled, and optical biopsy with AI-assisted ECS may avoid the need for forceps biopsy for endoscopists of any level.

Several recent studies have reported original CNNs in the diagnosis of GC. These were mainly divided into two categories: computer-aided detection (CADe) systems to focus on detection and computer-aided diagnosis (CADx) systems for optical biopsy. Miyaki et al. have developed a CADx system built by magnifying FICE images for classification between EGC and no-malignancy lesions. It showed an accuracy of 85.9%, sensitivity of 84.8%, and specificity of 87.0% [26]. In 2020, Horiuchi et al. have trained a CADx model with ME-NBI images of 1492 cases of EGC and 1078 cases of gastritis with sensitivity, specificity, PPV, and NPV of 95.4%, 71.0%, 82.3%, and 91.7%, respectively [27]. Similarly, Li et al. have reported that their CADx system with ME-NBI showed high sensitivity (91.2%), specificity (90.6%), and accuracy (90.1%) for the diagnosis of EGC [28]. Our result is comparable to that of these studies, and the AUC of our constructed CNN was 93.0%, which is satisfactory.

Several previous studies have reported a CADx system with ECS. In 2015, Mori et al. developed a CADx system for ECS imaging of colorectal lesions [32]. Recently, the same group constructed the EndoBRAIN system based on a large number of ECS images (69,142 images); they demonstrated that the EndoBRAIN system could distinguish neoplastic colon polyps from non-neoplastic colon polyps in ECS with NBI [33]. Maeda et al. have shown that the CNN could predict persistent histologic inflammation using ECS in patients with ulcerative colitis [34]. Kumagai et al. have developed their own AI system to analyze ECS images of the esophagus [35]. However, the application of the CNN with ECS in the stomach has not been examined. To the best of our knowledge, this is the first report to evaluate the performance of CNN with ECS to diagnose GC. In this study, we focused on intestinal-type EGC because GC has various histological types. Furthermore, most cases that needed to be differentiated from non-neoplastic lesions were at the early stage.

To determine the cause of misdiagnosis by the CNN, we analyzed ECS images with heatmaps (Fig. 3) in false-positive and false-negative cases. We can use heatmaps to visually verify the location at which the CNN is focused. Regarding the false-positive cases, the red area in the heatmap corresponded to the hyperchromatic cell or nuclei in the proper mucosal layer. These hyperchromatic cells, which exist outside lined foveolar epitheliums, may not be the nuclei of cancer cells, but inflammatory or mast cells. Observing the NGM with ECS, it is crucial to establish whether there is intestinal metaplasia. Methylene blue could be used to stain the intestinal metaplasia mucosa easily [36, 37], which develops the character of columnar absorptive cells and a brush border similar to that of the intestinal mucosa. Conversely, the foveolar epithelium on the surface of the fundic gland mucosa without intestinal metaplasia makes it difficult to stain using methylene blue. Therefore, when we observe fundic gland mucosa with active gastritis, not including intestinal metaplasia, other cells in the lamina propria are emphasized rather than the foveolar epithelium (Fig. 1k).

Figure 3g, h presents representative false-negative images for the CNN. The most common cause of false-negative cases was poor staining. Poor staining makes it difficult for the CNN to recognize nuclear shape, leading to misdiagnosis. In the present study, both endoscopists accurately diagnosed more than half of the false-negative images of the CNN (38/74 images), because the endoscopists can distinguish between the poorly stained region and evaluated region. Therefore, sufficient staining of the lesion is essential for adequate ECS diagnosis by the CNN. Most recently, an image-enhanced program named Texture and Color Enhancement Imaging (TXI) was developed, which allowed for remarkable color enhancement in ECS images, including in the poorly stained ones. Therefore, using TXI in ECS may increase the diagnostic performance of the AI-assisted ECS diagnosis system.

The CNN was inferior to the two experienced endoscopists in diagnostic sensitivity for EGC but demonstrated higher specificity in per-image analysis. Totally, the CNN was not superior to the endoscopists as for diagnostic ability. In contrast, when looking at the diagnosis time, it took > 20 min for the endoscopists to review all the images, whereas the CNN read all the images in 7 s. Moreover, our CNN for ECS has achieved a higher diagnostic ability than that achieved by the trainee; therefore, even trainees can accurately and easily establish ECS diagnoses using the CNN.

Our main concern is determining when and where to use this CNN. CADe systems for detecting a suspicious lesion for cancer require a higher sensitivity, whereas CADx for differentiating cancer from noncancer warrants a higher specificity. CAD for ECS is of the latter type. Considering the character of ECS, it is useful as a supportive diagnostic tool for the CNN to complement conventional endoscopic diagnosis when an endoscopic biopsy is not possible or pathological diagnosis is difficult to determine. In particular, we should use the CNN in patients who take three or more antithrombotic drugs and have lesions diagnosed as gastric indefinite for neoplasia [38]. 

This study had several limitations. First, this study was a retrospective single-center study, resulting in a small number of test images. Second, diffuse-type EGC, gastric adenomas, gastric polyps, and NGM with inflammatory cells, which contribute to poor-quality images, were not included in this study according to the exclusion criteria. Observation by ECS, especially in the stomach, is more likely to cause poor-quality images due to rich mucus cells leading to poor dye staining than observations by WLI, NBI, or ME-NBI. For clinical applications, these lesions and images should be included. Recently, Horiuchi et al. have investigated the usefulness of ECS with NBI in EGC diagnosis [39]. Therefore, further large-scale research, including ECS observation on other various gastric lesions and assessment on staining quality, is warranted in the future. Third, it is known that some noncancerous mucosa exists in cancer, and we cannot completely exclude the possibility that some of the images used in the training dataset as cancer ECS images are noncancer images. To exclude the false-positive contamination, we intended to focus on a cancerous part of a target cancer during ECS observation. We routinely performed white light and magnified NBI observation proceeding to ECS. Most of noncancerous parts in a cancerous lesion can be recognized with magnified NBI observation, and we may avoid the false-positive contamination during ECS observation of a target cancer. However, it is impossible to get a complete match between the histopathological image and ECS image and which may cause a decrease in the accuracy of the training images. Fourth, there may be a selection bias when sorting through all the images.

In summary, our CNN may be a useful CAD system in EGC diagnosis with higher specificity than diagnosis by endoscopists. Further investigation should be conducted for the construction of an AI-assisted system utilized for a wide variety of gastric lesions and diseases other than EGC.

## Data Availability

The datasets generated during the current study are available in the DRYAD repository, https://datadryad.org/stash/share/Cprw3eGjKiRb7l8lMiKQSKtiZDPt9bS0j9KuWEqsXBA

## Abbreviations

AUC: Area under the curve
CAD: Computer-aided diagnosis
CI: Confidence interval
CNN: Convolutional neural network
EGC: Early gastric cancer
ESD: Endoscopic submucosal dissection
FICE: Flexible spectral imaging color enhancement
GC: Gastric cancer
NBI: Narrow-band imaging
NGM: Noncancerous gastric mucosa
NPV: Negative predictive value
PPV: Positive predictive value
ROC: Receiver operating characteristic
WLE: White light imaging endoscopy
WLI: White light imaging
